# Supplementary material for: The Association of Nighttime Fasting Duration and Prostate Cancer Risk: Results from the Multicase-Control (MCC) Study in Spain
Source: Nutrients. 2021 Jul 30;13(8):2662. doi: 10.3390/nu13082662 (PMC8399976; doi:10.3390/nu13082662)
Supplement: Supplementary file 1 [file nutrients-13-02662-s001.zip › nutrients-1287639-Supplementary Material.pdf]

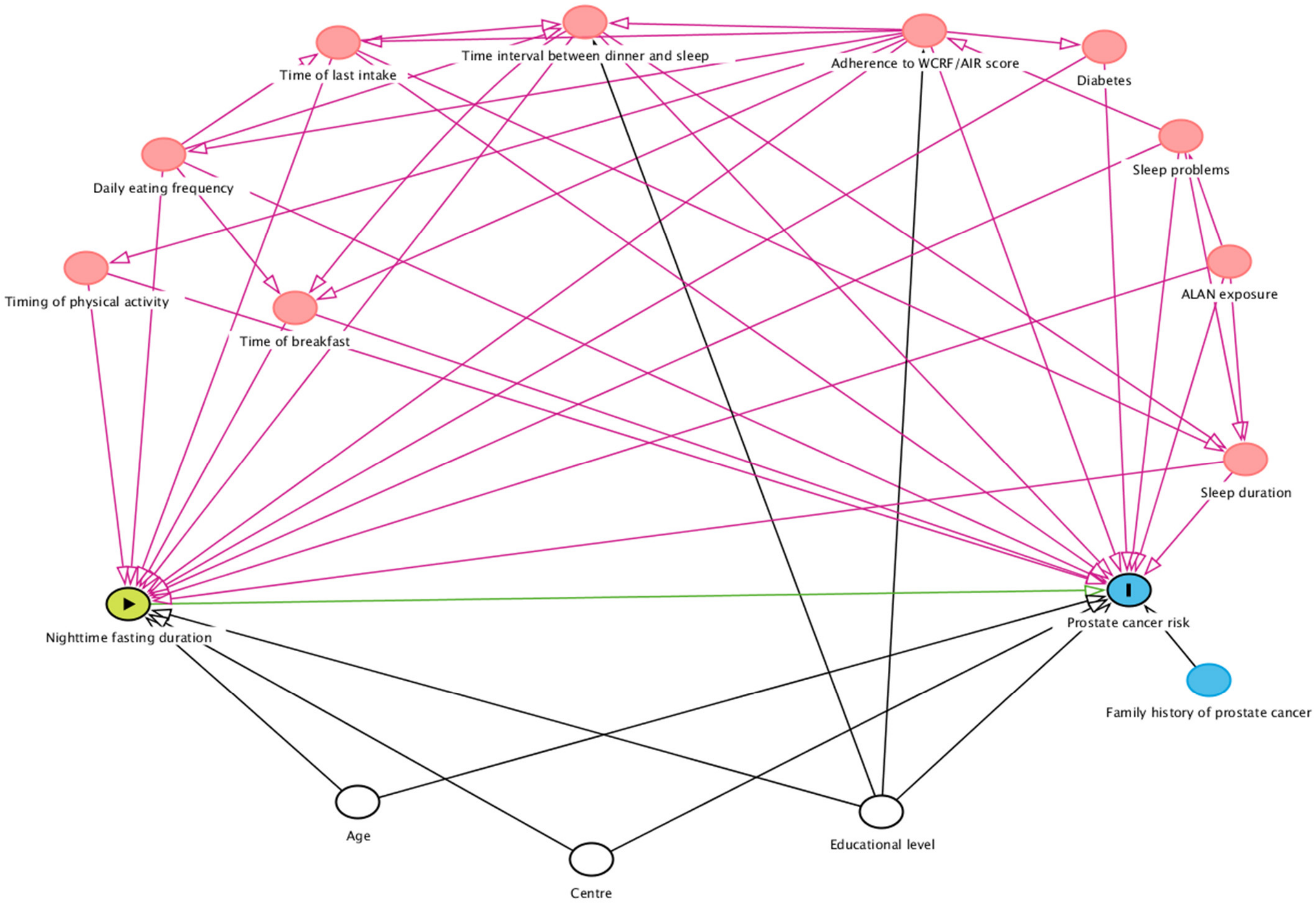

Figure S1. Directed acyclic graph representing the potential association between nighttime fasting duration and prostate cancer risk. White: adjusted variable; Pink: ancestor of exposure and outcome; Blue: ancestor of outcome. ALAN: Artificial light-at-night; WCRF/AICR = World Cancer Research Fund / American Institute for Cancer Research.

Table S1. Examination of further adjustment with potential confounders.

Variables	Controls (N= 848)	Cases (N= 607)	OR [a]	p-value [b]	OR [c]	OR [d]	% of change [e]
	Mean (SD) or N (%)	Mean (SD) or N (%)					
**Family history of prostate cancer**							
No	793 (93.5)	506 (83.4)	*Ref*		0.92 (0.73-1.16)	0.93 (0.74-1.18)	1%
Yes	55 (6.5)	101 (16.6)	2.86 (1.02-4.11)	**<0.001**			
**Chronotype**							
Morning	419 (50.5)	306 (50.5)	*Ref*		0.92 (0.73-1.17)	0.92 (0.73-1.17)	0%
Intermediate	303 (36.6)	224 (37.0)	1.10 (0.87-1.39)	0.437			
Evening	107 (12.9)	76 (12.5)	1.06 (0.75-1.49)	0.729			
Unknown	*19*	*1*					
**WCRF/AICR score**							
Low adherence [f]	307 (40.1)	203 (37.3)	*Ref*		0.86 (0.67-1.10)	0.88 (0.68-1.12)	2%
Medium adherence [g]	262 (34.2)	232 (42.6)	1.31 (1.01-1.70)	**0.043**			
High adherence [h]	196 (25.6)	109 (20.0)	0.83 (0.61-1.13)	0.244			
Unknown	*83*	*63*					
**Diabetes**							
No	671 (79.3)	518 (85.6)	*Ref*		0.92 (0.72-1.16)	0.92 (0.73-1.17)	0%
Yes	175 (20.7)	87 (14.4)	0.64 (0.47-0.85)	**0.002**			
Unknown	*2*	*2*					
**Indoor ALAN exposure**							
Total darkness	147 (17.4)	87 (14.4)	*Ref*		0.91 (0.72-1.15)	0.91 (0.72-1.16)	0%
Almost dark	348 (41.2)	211 (34.9)	1.02 (0.72-1.43)	0.920			
Dim light	261 (30.9)	204 (33.7)	1.24 (0.87-1.76)	0.239			
Quite illuminated	88 (10.4)	103 (17.0)	2.04 (1.35-3.08)	**<0.001**			
Unknown	*4*	*2*					

**Outdoor ALAN exposure**	0.01 (0.0)	0.01 (0.0)	NA	0.078	0.80 (0.60-1.06)	0.79 (0.59-1.05)	1%
Unknown	*327*	*251*					
**Sleep problems**							
No	577 (75.6)	454 (75.2)	*Ref*		0.85 (0.67-1.09)	0.85 (0.67-1.09)	0%
Yes	186 (24.4)	150 (24.8)	1.05 (0.81-1.35)	0.728			
Unknown	*85*	*3*					
**Sleep duration**							
7 hours or less	494 (65.1)	388 (64.7)	*Ref*		0.84 (0.66-1.07)	0.85 (0.67-1.08)	1%
More than 7 hours	265 (34.9)	212 (35.3)	1.08 (0.85-1.36)	0.528			
Unknown	*89*	*7*					
**Eating frequency**							
2 or less	26 (3.1)	11 (1.8)	*Ref*		0.92 (0.73-1.16)	0.94 (0.74-1.19)	2%
3	503 (59.3)	364 (60.0)	1.67 (0.82-3.63)	0.171			
More than 3	319 (37.6)	232 (38.2)	1.65 (0.80-3.64)	0.191			
**Time of first intake**							
8:30AM or before	466 (55.0)	314 (51.7)	*Ref*		0.92 (0.73-1.16)	0.79 (0.57-1.10)	**14%**
After 8:30AM	382 (45.0)	293 (48.3)	1.25 (0.89-1.76)	0.207			
**Time of last intake**							
10PM or later	294 (34.7)	227 (37.4)	*Ref*		0.92 (0.73-1.16)	0.96 (0.75-1.23)	4%
9:00 to <10PM	439 (51.8)	311 (51.2)	0.94 (0.74-1.19)	0.596			
Before 9PM	115 (13.6)	69 (11.4)	0.76 (0.52-1.10)	0.152			
**Interval between supper and sleep**							
≤ 1 hr	186 (22.2)	169 (28.1)	*Ref*		0.91 (0.72-1.15)	0.93 (0.74-1.18)	2%
From >1 to 2 hr	327 (39.0)	223 (37.0)	0.74 (0.56-0.97)	**0.031**			
> 2 hr	326 (38.9)	210 (34.9)	0.72 (0.54-0.96)	**0.028**			
Unknown	*9*	*5*					

**Daily caloric intake**	2030.6 (690.3)	2072.7 (670.8)	1.00 (1.00-1.00)	0.495	0.86 (0.67-1.10)	0.86 (0.67-1.10)	0%
Unknown	*83*	*63*					
**Timing of physical activity**							
Inactive [i]	54 (11.9)	48 (14.5)	*Ref*		1.05 (0.76-1.46)	1.06 (0.76-1.47)	1%
8-10AM	67 (14.8)	37 (11.2)	0.55 (0.30-0.99)	**0.049**			
10AM-12PM	68 (15.0)	67 (20.2)	1.03 (0.59-1.81)	0.903			
12PM-7PM	35 (7.7)	29 (8.8)	1.01 (0.52-1.97)	0.977			
7PM-11PM	75 (16.6)	46 (13.9)	0.71 (0.39-1.27)	0.247			
Any other pattern	153 (33.8)	104 (31.4)	0.75 (0.46-1.22)	0.244			
Unknown	*396*	*276*					

ALAN: Artificial light-at-night; N: sample size; NA: Not applicable; OR: odds ratio; SD: Standard deviation; WCRF/AICR = World Cancer Research Fund / American Institute for Cancer Research. All models were adjusted for age, centre and education. [a] Estimated OR for the association between each of the examined variables and prostate cancer risk. [b] p-value for the inclusion of each of the examined variables in the basic model. [c] Estimated OR for the association between nighttime fasting and prostate cancer risk (*Ref:* 11 hours or less vs. More than 11 hours). Participants with complete information on each of the examined variables. [d] For the association between nighttime fasting and prostate cancer risk including individually each of the variables (*Ref:* 11 hours or less vs. More than 11 hours). [e] Percentage of change of the OR for the association between nighttime fasting and prostate cancer risk after including each of the variables (OR [c] vs. OR [d]). [f] Men (0.25-3); Women (0.5-3.5). [g] Men (3.25-4); Women (3.75-4.25). [h] Men (4.25-6); Women (4.5-6). [i] Less than 1 MET * h/week as an annual average from 5 years of age onward with a lag of 1 year to the year of the interview.

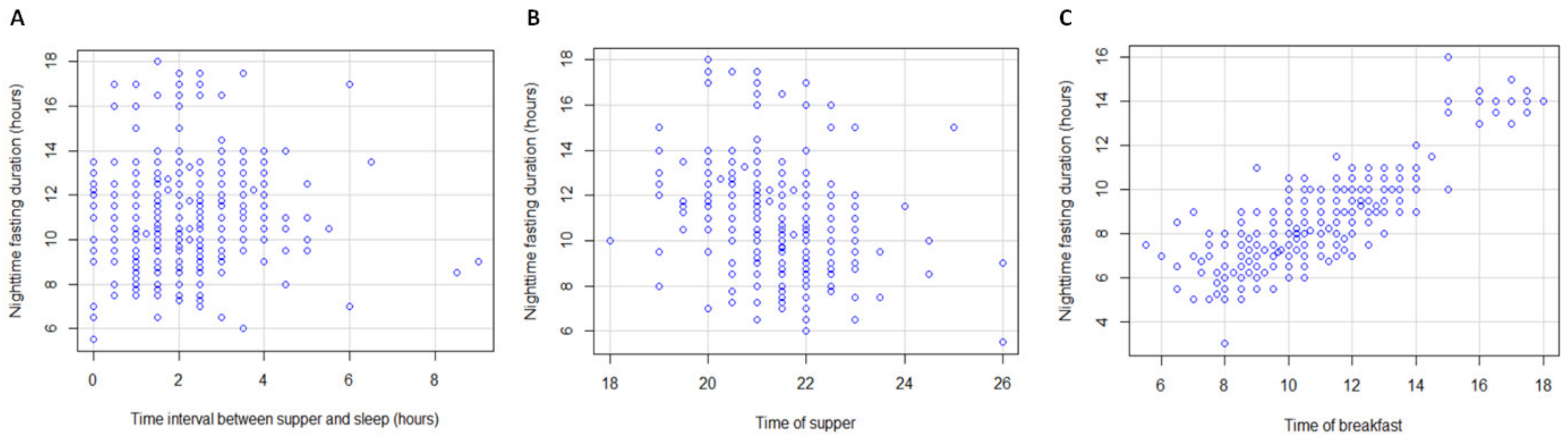

Figure S2. Scatter plot for each of the correlations among controls. **(A)** Correlation between nighttime fasting duration and interval between supper and sleep. Spearman's rank correlation rho = 0.12, p-value < 0.001. **(B)** Correlation between nighttime fasting duration and time of supper. Spearman's rank correlation rho = -0.36, p-value < 0.001. **(C)** Correlation between nighttime fasting duration and time of breakfast. Spearman's rank correlation rho = 0.83, p-value < 0.001

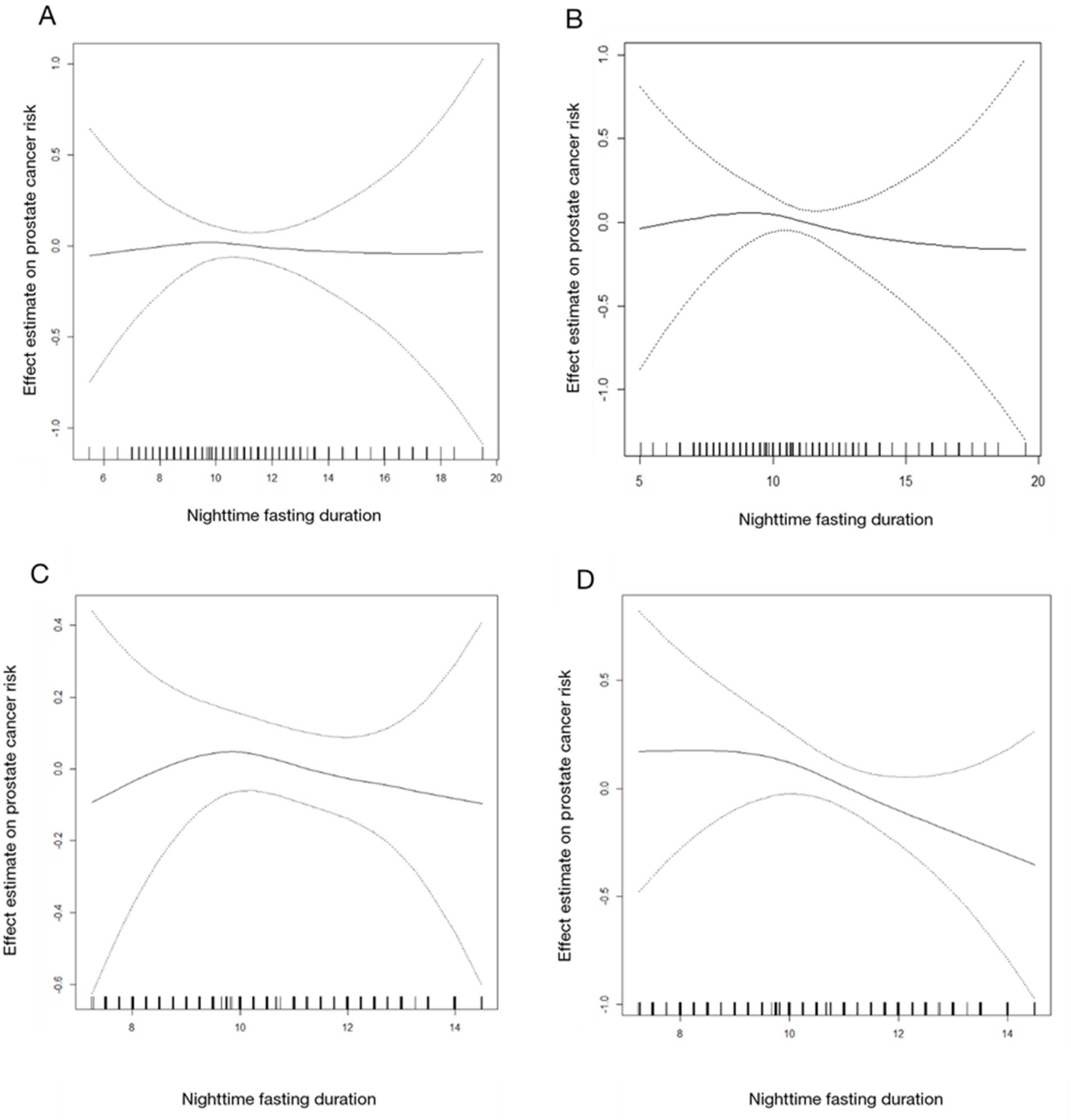

Figure S3. Generalized additive model examining the linearity of the association between nighttime fasting duration (continuous variable, in hours) and prostate cancer risk. We specified 2 degrees of freedom. **(A)** Model adjusted for age, center and educational level (test for linearity, p-value = 0.63). **(B)** Model adjusted for age, center, educational level and time of breakfast. **(C)** Model adjusted for age, center and educational level in dataset excluding outliers. **(D)** Model adjusted for age, center, educational level and time of breakfast in dataset excluding outliers.

Table S2. Association of nighttime fasting duration with prostate cancer risk. Exposure variable categorized into three levels based on the distribution of this variable in the control group

Nighttime fasting	Controls N (%)	Cases N (%)	OR (95% CI) [a]	OR (95% CI) [b]
≤10 hr.	289 (34.1)	219 (36.1)	*Ref*	*Ref*
(10 hr. – 12 hr.]	365 (43.0)	241 (39.7)	0.87 (0.67-1.12)	0.80 (0.60-1.06)
> 12 hr.	194 (22.9)	147 (24.2)	0.92 (0.68-1.24)	0.80 (0.54-1.18)

N = Sample size. OR = Odds Ratio; 95% CI = 95% Confidence Interval. [a] Adjusted for age, center and education. [b] Adjusted for age, center, education, diabetes (missing for 2 controls and 2 cases), indoor ALAN exposure (missing for 4 controls and 2 cases) and time of breakfast.

Table S3. Association of nighttime fasting and prostate cancer risk stratified by adherence to the WCRF / AICR score (missing for 83 controls and for 63 case)

Nighttime fasting	Controls N (%)	Cases N (%)	OR (95% CI) [a]	OR (95% CI) [b]
**Low adherence**				
≤ 11 hr.	164 (53.4)	109 (53.7)	*Ref*	*Ref*
> 11 hr.	143 (46.6)	94 (46.3)	0.93 (0.64-1.35)	0.73 (0.46-1.16)
**Medium adherence**				
≤ 11 hr.	154 (58.8)	146 (62.9)	*Ref*	*Ref*
> 11 hr.	108 (41.2)	86 (37.1)	0.78 (0.53-1.15)	0.65 (0.40-1.04)
**High adherence**				
≤ 11 hr.	111 (56.6)	60 (55.0)	*Ref*	*Ref*
> 11 hr.	85 (43.4)	49 (45.0)	0.95 (0.58-1.56)	0.76 (0.43-1.34)

N = Sample size; WCRF/AICR = World Cancer Research Fund / American Institute for Cancer Research. OR = Odds Ratio; 95% CI = 95% Confidence Interval. [a] Adjusted for age, center and education. [b] Adjusted for age, center, education, diabetes (missing for 2 controls and 2 cases), indoor ALAN exposure (missing for 4 controls and 2 cases) and time of first intake.

Table S4. Association of nighttime fasting duration with prostate cancer risk excluding outliers (N=58)

Nighttime fasting	Controls N (%)	Cases N (%)	OR (95% CI) [a]	OR (95% CI) [b]
≤ 11 hr.	466 (57.1)	335 (57.7)	*Ref*	*Ref*
> 11 hr.	350 (42.9)	246 (42.3)	0.90 (0.71-1.15)	0.76 (0.54-1.06)

N = Sample size. OR = Odds Ratio; 95% CI = 95% Confidence Interval. [a] Adjusted for age, center and education. [b] Adjusted for age, center, education, diabetes (missing for 2 controls and 2 cases), indoor ALAN exposure (missing for 4 controls and 2 cases) and time of breakfast.

**Table S5. Association of nighttime fasting duration with prostate cancer risk based on information on dietary habits the year before the circadian interview**				
**Nighttime fasting**	**Controls N* (%)**	**Cases N* (%)**	**OR (95% CI) [a]**	**OR (95% CI) [b]**
≤ 11.5 hr.	451 (52.1)	320 (51.9)	*Ref*	*Ref*
> 11.5 hr.	415 (47.9)	296 (48.1)	1.05 (0.84-1.31)	1.13 (0.88-1.46)

N = Sample size. OR = Odds Ratio; 95% CI = 95% Confidence Interval. [a] Adjusted for age, center and education. [b] Adjusted for age, center, education, diabetes (missing for 2 controls and 2 cases), indoor ALAN exposure (missing for 4 controls and 2 cases) and time of breakfast. *Number of cases and controls differ since we had different amounts of missing data in the exposure variable. With the 1y data 66 participants had missing data on nighttime fasting duration.

**Table S6. Prostate cancer relative risk by cancer subtypes with information on dietary habits the year before the circadian interview (missing for 17 cases)**				
**Nighttime fasting**	**Controls N (%)**	**Cases N (%)**	**RRR (95% CI) [a]**	**RRR (95% CI) [b]**
**Low aggressiveness**				
≤ 11.5 hr.	451 (52.1)	235 (53.0)	*Ref*	*Ref*
> 11.5 hr.	415 (47.9)	208 (47.0)	1.03 (0.81-1.31)	1.10 (0.83-1.46)
**High aggressiveness**				
≤ 11.5 hr.	451 (52.1)	76 (48.7)	*Ref*	*Ref*
> 11.5 hr.	415 (47.9)	80 (51.3)	1.13 (0.79-1.61)	1.23 (0.82-1.84)

N = Sample size. [a] Adjusted for age, center and education. [b] Adjusted for age, center, education, diabetes (missing for 2 controls and 2 cases), indoor ALAN exposure (missing for 4 controls and 2 cases) and time of breakfast. RRR = Relative Risk Ratio; 95% CI = 95% Confidence Interval. With the 1y data 66 participants had missing data on nighttime fasting duration.
